# Controlling mosquitoes with semiochemicals: a review

**DOI:** 10.1186/s13071-020-3960-3

**Published:** 2020-02-17

**Authors:** Madelien Wooding, Yvette Naudé, Egmont Rohwer, Marc Bouwer

**Affiliations:** 1 Department of Chemistry, University of Pretoria, Hatfield, Pretoria, South Africa; 2 Forestry and Agricultural Biotechnology Institute, University of Pretoria, Hatfield, Pretoria, South Africa

**Keywords:** Malaria, Vector mosquitoes, *Anopheles*, *Aedes*, *Culex*, Mosquito life-cycle, Semiochemicals, Chemical communication

## Abstract

The use of semiochemicals in odour-based traps for surveillance and control of vector mosquitoes is deemed a new and viable component for integrated vector management programmes. Over 114 semiochemicals have been identified, yet implementation of these for management of infectious diseases such as malaria, dengue, chikungunya and Rift Valley fever is still a major challenge. The difficulties arise due to variation in how different mosquito species respond to not only single chemical compounds but also complex chemical blends. Additionally, mosquitoes respond to different volatile blends when they are looking for a mating partner, oviposition sites or a meal. Analytically the challenge lies not only in correctly identifying these semiochemical signals and cues but also in developing formulations that effectively mimic blend ratios that different mosquito species respond to. Only then can the formulations be used to enhance the selectivity and efficacy of odour-based traps. Understanding how mosquitoes use semiochemical cues and signals to survive may be key to unravelling these complex interactions. An overview of the current studies of these chemical messages and the chemical ecology involved in complex behavioural patterns is given. This includes an updated list of the semiochemicals which can be used for integrated vector control management programmes. A thorough understanding of these semiochemical cues is of importance for the development of new vector control methods that can be integrated into established control strategies.
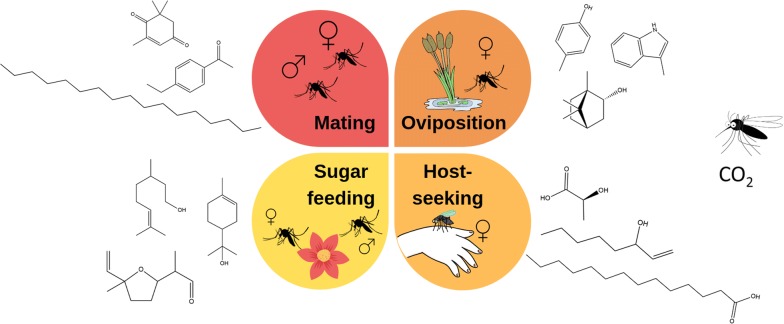

## Background

Mosquitoes transmit serious infectious diseases that include dengue, chikungunya, Rift Valley fever and malaria. In Africa, malaria remains as one of the most serious vector-borne diseases [1]. Malaria is spread by anopheline mosquitoes that transmit malaria parasites to humans. Of the hundreds of *Anopheles* species found worldwide, only a few dozen mediate the transmission of the *Plasmodium* parasite to humans [2]. Africa has over 128 native *Anopheles* mosquito species [3], with *An. gambiae* (*sensu stricto*), *An. coluzzii* and *An. funestus* being the predominant malaria vector species [4]. However, some minor species are also cause for concern. For example, the Asian native mosquito *An. stephensi* is now established in Ethiopia [4] and *An. arabiensis* is deemed a major cause of residual malaria transmission due to the species’ insecticide avoidance behaviours [2].

Vector control strategies such as indoor residual spraying (IRS) and long-lasting insecticidal net (LLIN) programmes have played a crucial part in the reduction of malaria cases between 2002 and 2017 [5–7]. There are two main problems affecting the future use of LLINs and IRS: first, these strategies used alone or combined will not eradicate malaria incidences in high transmission areas and secondly, insecticide resistance of the major malaria vectors in Africa is widespread and increasing [6, 7]. Another major concern with these vector control strategies is that these tools mainly target *Anopheles* vectors that feed and rest indoors and have a preference to feed at night. Changes in mosquito host preferences, time of feeding and an increase in outdoor feeding due to plasticity in mosquito behavioural responses have prompted the need for new and more environmentally friendly and robust vector control strategies that supplement current control strategies [5, 8]. The development and incorporation of novel vector control tools based on new scientific knowledge about mosquito behaviour and chemical ecology into integrated vector management (IVM) programmes are needed in order to reduce the burden and threat of mosquito-borne diseases [6].

Studying the intricate strategies that malaria mosquitoes have evolved to survive in their environment may result in novel control methods for malaria. Mosquito survival depends not only on how successful they are at finding suitable mating partners, oviposition sites and blood or sugar meals [9], but also on how effective they are at avoiding predators by moving around undetected and finding suitable shelter locations [10]. Mosquitoes can do this by detecting information from their environment through a set of sensitive sensory organs [10]. Artificially interfering with these processes may result in the ability to reduce mosquito populations and subsequently the incidence of malaria.

A range of environmental factors are detected by mosquitoes and are used as cues that can affect their behaviour. Changes that occur in the visual environment of the mosquito obviously play an essential role, but other factors such as fluctuations in the temperature or humidity levels also have an impact. Perhaps, the most important cues are those that are present in the volatile chemical environment that surrounds the mosquito [9]. Mosquitoes use odours to locate their hosts and it is known that different mosquito species depend on and detect different types of host odours [1, 11]. For example, *An. arabiensis* responds more strongly to carbon dioxide (CO_2_) as a general cue to find a host, while *An. gambiae* relies on other odours more specific to humans together with CO_2_ acting as a long-range attractant [11, 12]. Understanding how these odours differ and how they are perceived by specific mosquito species is an important field that has been well documented but is still not understood entirely.

New knowledge on the chemical ecology of insects (the study of chemical structures used by insects to mediate intra- and interspecific interactions) has led directly to the development of novel pest control strategies in agriculture and forestry [13]. Observations regarding the interactions of insects and their chemical environments together with the development of analytical techniques such as gas chromatography-mass spectrometry (GC-MS), high-performance liquid chromatography (HPLC), solid-phase microextraction (SPME) and electrophysiology, including electroantennography (EAG), single sensillum recordings (SSR) and gas chromatography coupled to electroantennographic detection (GC-EAD), has greatly facilitated research in the field of chemical ecology [14]. Chemical ecology of mosquitoes, specifically how mosquito behaviour is mediated by odour (Table 1), is a major field of study that aims at improving control strategies that depend on synthetic odour lures which are popular for use in lure-and-kill strategies [9].

**Table 1:** List of identified semiochemicals used by mosquitoes during mating, oviposition, host-seeking and sugar feeding

Mosquito species	Semiochemicals involved in key behavioural strategies of mosquitoes			
	Mating	Oviposition	Host-seeking	Sugar feeding
*Ae. aegypti*	2,6,6-Trimethylcyclohex-2-ene-1,4-dione (ketoisophorone)^a^ [26]	Phenol [11, 40]	CO_2_ [11, 19, 61]	Terpineol [96]
	2,2,6-Trimethylcyclohexane-1,4-dione^a^ [26]	*p*-Cresol [40]	Lactic acid [11]/(*S*)-lactic acid [9, 19]	Geraniol [96]
	1-(4-Ethylphenyl) ethanone^a^ [26]	3-Methylindole (skatole) [40]	(*R*)-1-Octen-3-ol [69, 111]	Eugenol [96]
	CHs^b^: Me-C_29_, *n*-heptadecane, *n*-pentacosane, *n*-hexacosane^c^ [24]	4-Ethylphenol (stimulant) [40]	Hexanoic acid [69]	Citral [96]
		Camphor (stimulant) [40]	2-Compound blend: octanal and nonanal [69]	Citronellol [96]
		β-pinene (stimulant) [40]	Geranyl acetone [69, 72]	Fatty acids [96]
		Borneol (stimulant) [40]	6-Methyl-5-hepten-2-one [69]	Amyl acetate [96]
		Borneol acetate (stimulant) [40]	Linalool [69, 72]	Toluene [96]
		3-compound blend: nonanoic acid, tetradecanoic acid and methyl tetradecanoate [53]	4-Compound blend: heptanal, octanal, nonanal and decanal [112]	Phenylethyl alcohol [96]
		3-compound blend: 3-methylindole, *p*-cresol and phenol [59]	Sulcatone [72]	Phenylacetaldehyde [96]
		CHs [24]	Dodecanal [72]	Lilac aldehyde [96]
		6-hexanolactone [55]	Limonene [72]	(*Z*)-3-Hexenyl acetate [96]
		Methyl dodecanoate [55]	2-Ethyl hexanol [72]	Linalool oxide [96]
		Dodecanoic acid [55]	Butyric acid [72]	Linalool [96]
		Methyl tetradecanoate [55]	Heptanoic acid [72]	Benzaldehyde [96]
		Tetradecanoic acid [55]	Octanoic acid [72]	Lilac alcohol [96]
		Methyl (*Z*)-9-hexadecenoate [55]	Nonanoic acid [72]	Acetophenone [96]
		Methyl hexadecanoate [55]		Methyl salicylate [96]
		(*Z*)-9-Hexadecenoic acid [55]		Hexanal [96]
		Hexadecanoic acid [55]		1-Hexenol [96]
		Methyl-(*Z*)-9-octadecenoate [55]		(*Z*)-3-Hexen-1-ol [96]
		Methyl octadecenoate [55]		Benzenoids [104]
		(*Z*)-9-Octadecenoic acid [55]		20-Compound blend: butanoic acid^d^, 2-methylpropionic acid^d^, 2-methylbutanoic acid^d^, 3-methylbutanoic acid^d^, benzoic acid^d^, hexanoic acid^d^, (−)-α-pinene^d^, (−)-β-pinene, (−)-sabinene, (E/Z)-ocimene, germacrene-D, benzaldehyde^d^, acetophenone^d^, artemisia ketone, umbellulone, (Z)-3-hexenyl acetate, hexyl acetate, yomogi alcohol, phenyl-2,3-butanedione, 3-hydroxy-4-phenyl-2-butanone [103]
		Octadecanoic acid [55]		Nonanal [102]
		*n*-Heneicosane [56]		
		Propyl octadecanoate [58]		
*Ae. albopictus*	–	3-Methylindole (skatole) [11, 40]	CO_2_ [11, 19, 61]	–
		*p*-Cresol [40, 41]	1-Octen-3-ol [11]	
		CHs [24]		
*Ae. triseriatus*	–	*p*-Cresol [37, 40]	CO_2_ [11, 19, 61]	–
		*o*-Cresol [40]	1-Octen-3-ol [11]	
		4-Methylcyclohexanol [40]		
		2,4-Dimethylphenol [40]		
		2,3-Dimethylphenol [40]		
		4-Ethylphenol [40]		
*An. gambiae*	CHs: Me-C_29_, Me-C_30_, Me-C_31_^c^ [24]	3-Methylindole (skatole) [41]	CO_2_ [11, 19, 61]	Hexanal [94]
		Cedrol [46]	Octanal [113]	β-Pinene [94]
			Decanal [113]	Limonene [94]
			2-Nonanone [113]	β-Ocimene [94]
			Benzothiazole [113]	(*E*)-Linalool oxide [94]
			2-[(2-Ethylhexyl)oxy]-ethanol [113]	(*E*)-β-Farnesene [94]
			5-Compound blend with butan-1-amine, 2-pentadecanone and 1-dodecanol [114]	3-Compound blend: (*E*)-linalool oxide, β-pinene, β-ocimene [115]
			(*E*)-3-Methyl-2-hexenoic acid [84]	
			(*Z*)-3-Methyl-2-hexenoic acid [84]	Sesquiterpenes and alkenes [104]
			7-Octenoic acid [84]	
			Acetone [11]	
			Lactic acid [11]/(*S*)-lactic acid [9, 19]	
			1-Octen-3-ol [11]	
			4-Methylphenol [11]	
			Aliphatic carboxylic acids [11]	
			Carboxylic acids [9]	
			Ammonia [9]	
			3-Compound blend: ammonia, (*S*)-lactic acid and tetradecanoic acid [1, 90, 91]	
			5-Compound blend [the Mbita blend (MB)]: butan-1-amine, 3-methyl-1-butanol, ammonia, (*S*)-lactic acid and tetradecanoic acid [1, 66]	
			3-Methyl-1-butanol [92] 2-Butanone (as CO_2_ substitute) [71]	
			4-Compound blend: heptanal, octanal, nonanal and decanal [115]	
			3-Compound blend: heptanal, nonanal and octanal [82]	
*An. arabiensis*	CHs: *n*-hentriancontane, Me-C_29_, Me-C_30_, Me-C_31_^c^ [24]	8-Compound blend: ß-caryophyllene, decanal, sulcatone (6-methyl-5-hepten-2-one), limonene, 3-carene, ß-pinene and α-pinene [49]	CO_2_ [11, 19, 61]	Cyclic and bicyclic Monoterpenes [97]
		3-Compouond blend: benzaldehyde, nonanal and (*1R*)-(+)-α-pinene [50]	5-Compound blend: butan-1-amine, 3-methyl-1-butanol, ammonia, (*S*)-lactic acid and tetradecanoic acid [66]	
		4-Compound blend: benzaldehyde, nonanal, (1R)-(+)-α-pinene and *p*-cymene [50]	2-Butanone (as CO_2_ substitute) [71]	
			5-Compound blend with 1-dodecanol [114]	
*An. funestus*	–	–	CO_2_ [11, 19, 61]	–
			5-Compound blend: butan-1-amine, 3-methyl-1-butanol, ammonia, (*S*)-lactic acid and tetradecanoic acid [66]	
			2-Butanone (as CO_2_ substitute) [71]	
			5-Compound blend with 2-pentadecanone [114]	
*An. stephensi*	–	CHs: C_21_-fatty acid ester, propyl octadecanoate [58]	CO_2_ [11, 19, 61]	–
			Acetone [11]	
			1-Octen-3-ol [11]	
*An. coluzzii*	–	Dimethyl disulfide (DMDS) [116]	CO_2_ [11, 19, 61]	–
		Dimethyl trisulfide (DMTS) [116]	(*R*)-1-Octen-3-ol [111]	
		6-Methyl-5-hepten-2-one (sulcatone) (repellent) [116]		
*Cx. quinquefasciatus*	CHs^c^ [24]	*Erythro*-6-acetoxy-5-hexadecanolide (*Culex* oviposition pheromone) [42–44]	CO_2_ [11, 19, 61]	–
		5-Compound blend: phenol, 4-methylphenol. 4-ethylphenol, indole and 3-methylindole (skatole) [11, 51]	(*E*)-2-Decenal [85]	
		Skatole [40, 41]	Undecanal [85]	
		Acetaldehyde [60]	Dodecanal [85]	
		*p*-cresol [40]	Tertradecanal [85]	
		2-tridecanone [40]	Pentadecanal [85]	
		Indole [40]	Hexadecanal [85]	
		Trimethylamine [40]	Heptadecanal [85]	
		Nonanal [40]	Octadecanal [85]	
			4-Compound blend: CO_2_, acetone, 1-octenol-3-ol and butonoic acid [9].	
			1-Octen-3-ol [11]/ (R)-1-octen-3-ol [111]	
*Cx. tarsalis*	CHs^c^ [24]	*Erythro*-6-acetoxy-5-hexadecanolide [11, 45]	CO_2_ [11, 19, 61]	–
		*p*-cresol [40]	1-Octen-3-ol [11]	
		Indole [40]		
		Skatole [40]		
		Dimethyltrisulfide [40]		
		Phenol [40]		
		Nonanal [40]		
		Naphthalene (stimulant) [40]		
*Cx. pipiens*	CHs^c^ [24]	–	CO_2_ [11, 19, 61]	Terpineol [96]
			Lactic acid [11]	Geraniol [96]
				Eugenol [96]
				Citral [96]
				Citronellol [96]
				Fatty acids [96]
				Amyl acetate [96]
				Toluene [96]
				Phenylethyl alcohol [96]
				Phenylacetaldehyde [96]
				Lilac aldehydes [96]
				(*Z*)-3-Hexenyl acetate [96]
				Linalool oxide [96]
				Linalool [96]
				Benzaldehyde [96]
				Lilac alcohol [96]
				Acetophenone [96]
				Methyl salicylate [96]
				Hexanal [96]
				1-Hexenol [96]
				(Z)-3-Hexen-1-ol [96]
				Benzenoids [104]
				20-Compound blend: butanoic acid^d^, 2-methylpropionic acid^d^, 2-methylbutanoic acid^d^, 3-methylbutanoic acid^d^, benzoic acid^d^, hexanoic acid^d^, (−)-α-pinene^d^, (−)-β-pinene, (−)-sabinene, (E/Z)-ocimene, germacrene-d, benzaldehyde^d^, acetophenone^d^, artemisia ketone, umbellulone, (Z)-3-hexenyl acetate, hexyl acetate, yomogi alcohol, phenyl-2,3-butanedione, 3-hydroxy-4-phenyl-2-butanone [103]

^a^Aggregation pheromone

^b^CHs: cuticular hydrocarbons

^c^Contact pheromones

^d^Human shared semiochemicals used during sugar feeding

**Fig. 1:**
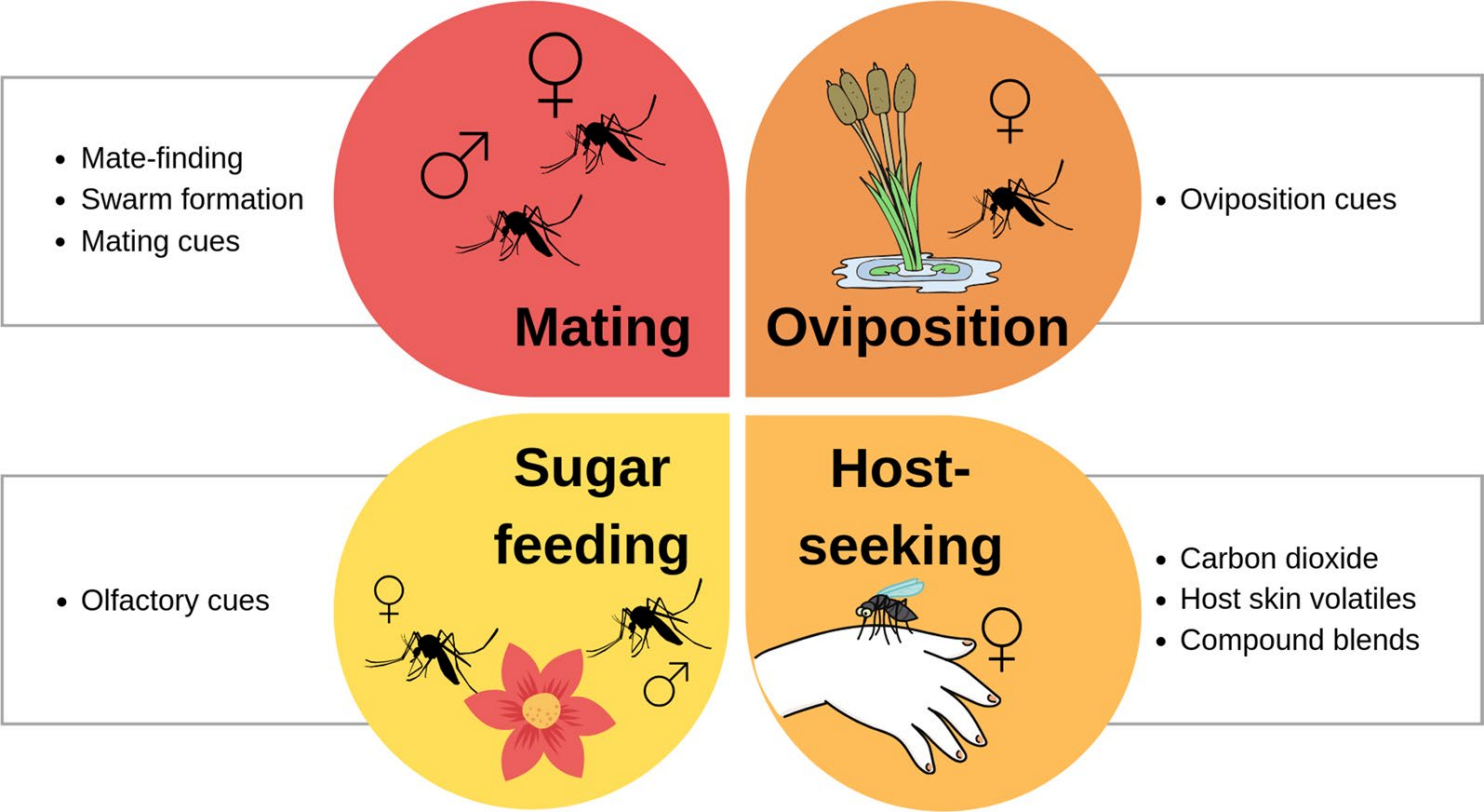
An overview of the key behavioural patterns, influenced by semiochemicals, discussed in this review. The four main behaviours that are targeted include mating, oviposition, host-seeking and sugar feeding

This literature review discusses chemical communication in vector mosquitoes, with the focus on malaria vectors. Key chemical cues and signals affecting different behavioural patterns in the adult mosquito are discussed (Fig. 1). The emphasis is on providing an updated list of chemical attractants which can be used in IVM programmes. Known semiochemicals for eleven mosquito species are tabulated.

## Semiochemicals as identified during the different mosquito life-cycle stages

Chemical cues and signals mediate key behaviours during the different stages in the life-cycle of an adult mosquito [15, 16]. Signals are produced for the specific purpose of changing the behaviour of an individual of a species, whereas cues are not produced for any specific purpose, but they are detected by individuals and can lead to behavioural responses [17]. Host finding by the adult mosquito occurs through a series of behavioural steps that are influenced by external chemical cues. A mosquito can be primed for activity by a specific chemical or blend produced by a potential host. This is followed by the initiation of flight to bring the insect within the vicinity of the source [18]. The mosquito will then continue to respond to external stimuli during flight for orientation through optomotor anemotaxis (movement in response to air currents and visual stimuli). Visual cues, such as contours against a background, that aid the mosquito during upwind flight and physical cues, such as convection heat and body moisture, play a role during host seeking. These cues, together with chemical cues, influence the orientation of a mosquito towards the host and can induce a landing response [11, 12, 18]. The individual importance of chemical, visual and physical cues that impact on the mosquito’s flight behaviour is well studied. However, the mechanism of how these cues work together to result in specific types of behaviours remains a topic that requires further investigation.

Another major challenge in identifying mosquito semiochemicals is the degree of plasticity that is observed in the behavioural responses of mosquitoes. The internal physiological state of the mosquito will alter the behavioural response elicited by a chemical cue or signal [9, 11, 19]. For example, many mosquito species have an inherent preference for a specific host (e.g. a preference for birds or mammals). This preference is easily countermanded by internal physiological factors, such as the level of starvation, and by external factors such as host availability [11, 19]. If there is an urgent need to feed, then the mosquito may settle for hosts that are normally not preferred. The physiological state of the mosquito will thus influence its response to certain behavioural cues. This state varies depending on the age, size, nutritional status and gonotrophic stage of the mosquito. Once the optimal internal state for a specific reaction is met then the mosquito will respond in the appropriate manner to the correct stimulus [11]. This plasticity in mosquito behaviour can impede the identification of important mosquito semiochemicals because the species being studied needs to be in the correct state to respond. This adds to the analytical challenge of correctly identifying semiochemicals that mediate certain behaviours.

### Mating behaviour

Semiochemicals are known to be involved in the mating behaviour of mosquitoes. There are different behavioural patterns that occur at different mating stages and these also vary for different species [11, 20]. Mating behaviour includes maturation feeding (feeding required for an insect’s gonads to mature for gamete production) followed by swarm formation, in some species, and a sequence of mate-finding behaviours that are guided by a set of chemical mating cues and signals [11]. Mating cues and signals are important to ensure the survival of a species and these cues and signals may help isolate a species, especially if they are unique to that species. Different, but related species that co-occur in the same environment sometimes have very similar odour cues and signals and, in such cases, isolation occurs by having mating period at different times of the day [21, 22]. This mechanism serves to prevent interspecies hybridisation [11]. Volatile chemicals that form part of the signals and cues used during these behaviours are thus not the same for the different species and need to be studied separately if specific control applications are to be developed. In this section a concise review of current insights into mate finding, swarm formation and mating cues with the emphasis on semiochemicals will be discussed.

#### Mate-finding

Chemical cues and signals are used by both male and female mosquitoes to find a conspecific mating partner. This behaviour is crucial for mosquito reproductive success rates [11]. Differences exist between species regarding the type of chemical compounds that they use. In some species, females need to locate male swarms and they do this by using olfactory cues and perhaps the same visual cues that guide male mosquitoes to form swarms [20]. While in other species males find foraging females by following kairomones, chemicals involved in mosquito-host interactions, that are indicative of the presence of a female during her blood-feeding behaviour [11, 20]. During this process, female mosquitoes are first attracted to a host, be it animal or human, through the kairomones that the host produces in breath, sweat or skin emanations [14]. Male mosquitoes, of certain species, then locate blood-feeding females by following the kairomone trail [11, 14]. The behaviour of a male mosquito intercepting a female near a host is known to occur for *Aedes* spp. and *Mansonia* spp. of the Culicidae [11]. Hapairai et al. [23] reported collecting a larger number of male than female *Ae. aegypti* when using human bait collections. This finding shows that the males of some species are attracted to and orientate towards host cues in the field [23].

Correctly identifying kairomones for the control of mosquitoes is possible but it may be that effective blends are different for each of the sexes. Furthermore, the challenge lies not only in identifying these chemicals but also to realise that differences exist for different species. Close-range species recognition cues that ultimately allow male mosquitoes to correctly recognise their conspecific females could potentially be useful as a variety of mosquito species will likely be present near a host [11]. The identification of a species-specific sex pheromone that is useful for selective control of vector mosquito species remains elusive. The existence of such pheromone can pave the way towards selective control of vector mosquitoes: the ultimate goal.

#### Swarm forming

It is proposed that both male and female mosquitoes produce aggregation pheromones that attract both sexes to the mating swarm [24]. How females locate male swarms is still unclear. Females may use similar visual cues (i.e. swarm markers) as males and olfactory cues, such as aggregation pheromones, to locate swarms [11, 20]. Laboratory studies have shown that when swarming *Ae. aegypti* males or females are placed upwind in an olfactometer a flight response is elicited in female mosquitoes, suggesting that both female and male *Ae. aegypti* produce a volatile attractant as one of many signals to initiate swarm formation [14, 24, 25]. Fawaz et al. [26] have identified 2,6,6-trimethylcyclohex-2-ene-1,4-dione (ketoisophorone), 2,2,6-trimethylcyclohexane-1,4-dione (the saturated analogue of ketoisophorone) and 1-(4-ethylphenyl) ethanone as aggregation pheromones for *Ae. aegypti*. These compounds may be the same or structurally related to aggregation pheromones that related mosquito species use and should be evaluated further.

Host odours also play a role in aggregation and mating as many mosquito species mate near their host [20]. For example, *Ae. albopictus* form small swarms of 3 to 40 individuals around the ankles and feet of human hosts and *Ae. aegypti* has been observed to do the same [14, 24]. A study by Cabrera and Jaffe [25] revealed that *Ae. aegypti* males can be induced to form swarms in response to host volatiles. Although the above-mentioned studies have provided insights into how swarms are formed through host odours, more studies are needed to conclusively show evidence for the existence of an aggregation pheromone. These studies should also consider the combined influence of visual and chemical cues on swarm formation. Such studies will be essential for the elucidation of the cause of the aggregation behaviour and for finding evidence of the existence of aggregation pheromones for other mosquito species [24].

#### Mating cues

The use of contact sex pheromones in dipterans for species recognition during mating is well documented, leading to the assumption that this mode of species recognition is also utilised by mosquitoes [14, 24]. Observations in support of sex pheromones for mosquitoes include studies done on adult male crabhole mosquitoes (*Deinocerites cancer*) [27] and winter mosquitoes (*Culiseta inornata*) [28] that revealed the male’s ability to locate female pupae to mate with newly emerged virgin female mosquitoes [27]. Mating has also been perceived between males and empty pupal cases or recently dead females for winter and crabhole mosquito species [27, 28]. *Culiseta inornata* males have been observed touching conspecific mates with their front leg tarsi [28]. Furthermore, a decrease in mating attempts in *Ae. albopictus* males have been recorded when their terminal tarsomeres of the prothoracic and mesothoracic tarsi were treated with a solvent mixture [14, 24, 29]. These studies found that short-range contact pheromones are most probably used by male and female mosquitoes in a swarm to recognise their own species. The volatility of aggregation pheromones makes these unlikely candidates for close range species recognition inside the swarm. This is due to the dynamic nature of swarms, specifically mixed species swarms [30].

Although cuticular hydrocarbons (CHs) normally act as a desiccation barrier for insects they are potential contact pheromones for some mosquito species. It is possible that low volatility contact pheromones play an important part in species recognition, especially when a range of other mosquito species are present near a host [11, 14]. Female *Cx. quinquefasciatus* Say, *Cx. tarsalis* Coquillett and *Cx. pipiens* L. are attracted to benzene extracts of conspecific males indicating that the extracted CHs may act as pheromones during mating [24]. Cuticular hydrocarbon profiles appear to be different between mated and unmated *An. gambiae* (*s.s.*) and *Ae. aegypti* females. In *Ae. aegypti*, the ratio of *n*-heptadecane, *n*-pentacosane and *n*-hexacosane is altered drastically after mating [24]. Such changes could potentially serve to fingerprint females that are already mated.

Qualitative differences in CH profiles between the two sexes of *An. gambiae* and age-dependent changes of the CH profiles of the different sexes suggest that CHs are possible cues for mate choice and sex recognition [24, 31]. Hydrocarbon analysis has also been used to discriminate between closely related mosquito species or populations and this tool is useful for discriminating morphologically similar species such as *An. gambiae* and *An. arabiensis* [24]. Qualitative differences in CH profiles, specifically *n*-hentriacontane (Me-C_31_), *n*-nonacosane (Me-C_29_) and *n*-triacontane (Me-C_30_), are used to discriminate between these sympatric species and Me-C_29_ has been identified as a pheromone candidate for *Ae. aegypti* [24, 32–34]. Mosquito hydrocarbons might consequently not only be important for species differentiation in order to obtain vital epidemiological data for vector control, but they are also potential sex pheromones which can be used in future lure-and-kill control strategies [24]. The challenge lies in how exactly these short-range chemical cues may be used in vector control strategies. The most likely strategy is with the interference of the mosquito’s landing response.

The importance of acoustic cues and wingbeat frequency in mosquito mating behaviour has been documented by various researchers and should not be overlooked when implementing vector control strategies during mating [14] (reviewed by Gibson, Warren & Russell [35]). The unique properties of the mosquito antennae and Johnston’s organ, which is located at the base of each antenna, allow mosquitoes to be more sensitive to sound than any other insect [35].

### Oviposition

The selection of an oviposition site for many mosquito species is determined by visual and chemical cues [11]. Chemical cues, produced by conspecifics, used as oviposition attractants include egg raft, larval and pupal pheromones [11, 36]. The presence of conspecific larvae and their associated semiochemicals at oviposition sites indicates suitable conditions for larval development [36]. Breakdown products, in natural water bodies, of bacterial origin are also used as chemical cues for oviposition [11]. The chemical ecology and oviposition behaviour of gravid mosquitoes, including attractants and deterrents such as water salinity, phytochemicals and insecticides, was comprehensively reviewed by Bentley and Day [37]. An updated list and further research on oviposition attractants for mosquito larvae control is needed as targeting mosquito larvae is deemed a more successful long-term approach to vector control. Targeting larvae removes vectors before they can reproduce and transmit diseases [38]. This section highlights the influence of semiochemicals, singular or blends of chemical compounds, on oviposition site selection as well as egg-laying behaviour in mosquitoes.

#### Oviposition cues

Reviews by Navarro-Silva et al. [39] and Afify and Galizia [40] highlight the importance of semiochemicals that mediate oviposition in mosquitoes. Odour attractants involved in oviposition site selection have been identified from plant material, faeces from mosquito larvae as well as secretions from mosquito larvae, pupae and eggs [41, 42]. The first breakthrough on the use of chemical cues for oviposition site selection was with the identification of the oviposition pheromone, *erythro*-6-acetoxy-5-hexadecanolide, which was extracted from the apical droplet at the tip of the eggs of *Cx. quinquefasciatus* by Laurence and Pickett [43, 44]. Studies reviewed by Takken and Knols [11] have shown that gravid conspecifics, as well as *Cx. tarsalis*, are highly attracted to the *erythro*-6-acetoxy-5-hexadecanolide, and electrophysiological activity has also been demonstrated by *Cx. quinquefasciatus* in response to this pheromone [45]. Another major development on oviposition odour attractants was achieved by Lindh et al. [46] during a study conducted in Kenya which identified the first oviposition attractant for gravid female *An. gambiae*, namely cedrol. A follow-up study, conducted by Eneh et al. [47], on the natural source of the chemical, found that cedrol is produced by fungal species found on grass rhizomes near the natural mosquito breeding site. This brought to light the influence of both microbes and vegetation for oviposition site selection in mosquitoes.

The influence of grass volatiles on oviposition site selection was further demonstrated by Asmare et al. [48], as blood-fed *An. arabiensis* females showed an attraction to these volatiles; the volatiles in question are yet to be identified. The importance of vegetation influencing oviposition site selection was highlighted by Wondwosen et al. [49] in a study which revealed that rice volatiles attract gravid *An. arabiensis* females. Eight compounds, namely ß-caryophyllene, decanal, sulcatone (6-methyl-5-hepten-2-one), limonene, 3-carene, ß-pinene, and α-pinene, elicited antenna responses during GC-EAD experiments. Furthermore, the study revealed that a complete blend of the compounds, released at the lowest effective dose (10 ng), was needed to induce the full behavioural response of attraction and oviposition [49]. Sugarcane pollen-associated volatiles attracted and induced egg laying in *An. arabiensis* females during a still air two-port olfactometer bioassay. Two blends were identified as being attractive for gravid *An. arabiensis* females, namely a three compound synthetic blend of benzaldehyde, nonanal and (*1R*)-(+)-α-pinene and a four compound synthetic blend of benzaldehyde, nonanal, (*1R*)-(+)-α-pinene and *p*-cymene [50]. Blend composition appears to be critical for eliciting the oviposition behaviour.

The presence of bacteria at oviposition sites has also been shown to impact the selection of suitable oviposition sites. *Culex quinquefasciatus* was found to be attracted to a five-compound blend, composed of phenol, 4-methylphenol, 4-ethylphenol, indole, and 3-methylindole (skatole), produced by bacteria found in hay infused waterbodies that are used as oviposition sites [11, 51]. In addition, olfactory responses with egg-laying behaviour in *Culex* mosquitoes have been found to be induced by the oviposition site volatile 3-methylindole [52]. Electrophysiological studies done revealed that *Ae. albopictus* responds to 3-methylindole and *Ae. aegypti* respond to phenol [11]. Field studies done in Tanzania revealed a synergistic oviposition response of *Cx. quinquefasciatus* to 6-acetoxy-5-hexadecanolide blended with either 3-methylindole or volatiles produced by bacteria found in soakage pit water [11]. Bacterial kairomones have been shown to affect oviposition in *Ae. aegypti* in a dose dependent manner. A nonanoic acid (16%), tetradecanoic acid (83%) and methyl tetradecanoate (1%) blend was found to stimulate oviposition, whereas hexadecenoic acid methyl ester deterred oviposition [53]. The importance of bacterial profiles in combination with physiochemical properties and semiochemicals for oviposition site selections was demonstrated by Eneh et al. [54] in a study showing that on average twice as many *An. arabiensis* instar larvae were found in freshwater ponds when compared to aged ponds (4-days-old). This indicates that volatiles of bacterial origin play a crucial role for oviposition site selection.

Odour cues originating from mosquito eggs or larvae can potentially be as used as attractants or repellents in vector control strategies. Cuticular hydrocarbons of mosquito eggs play a vital role in preventing egg desiccation and have been investigated as a potential source of oviposition pheromones. Cuticular hydrocarbons were listed as oviposition pheromones for *Ae. aegypti* and are known behavioural modifiers at breeding sites for *Ae. albopictus* [24]. Ganesan et al. [55] identified compounds that induce an ovipositional response in gravid female *Ae. aegypti*. These compounds included 6-hexanolactone, methyl dodecanoate, dodecanoic acid, methyl tetradecanoate, tetradecanoic acid, methyl (Z)-9-hexadecenoate, methyl hexadecanoate, (Z)-9-hexadecenoic acid, hexadecanoic acid, methyl (Z)-9-octadecenoate, methyl octadecanoate, (Z)-9-octadecenoic acid, and octadecanoic acid. A significant positive ovipositional response was obtained in gravid female *Ae. aegypti* with dodecanoic and (Z)-9-hexadecenoic acids whereas all the esters investigated induced a deterrent/repellent ovipositional effect [55]. Mendki et al. [56] identified *n*-heneicosane as an oviposition attractant pheromone from the larval cuticle of *Ae. aegypti*. The dose-dependent effect of *n*-heneicosane was investigated by Seenivasagan et al. [57] using electroantennogram (EAG) techniques. These authors showed that EAG response increased with an increasing stimulus concentration and behavioural repellence was reported for concentrations above 50 ppm [57]. Dose-dependent EAG responses from the antenna of gravid female *Ae. aegypti* and *An. stephensi* mosquitoes were recorded for the C_21_-fatty acid ester, propyl octadecanoate [58].

The significance of compound blends, rather than single impact compounds, responsible for attracting gravid mosquitoes has gained more attention in recent literature as is evident from the aforementioned studies. The use of compound blends in vector control strategies targeting oviposition was demonstrated in a study by Baak-Baak et al. [59] where a blend of synthetic chemicals, *viz* 3-methylindole, *p*-cresol and phenol, induced a response in gravid *Ae. aegypti* female mosquitoes. The authors demonstrated that an ovitrap baited with a blend of certain attractants can be used to monitor and potentially control mosquito populations [59]. The synergistic and/or antagonistic oviposition response of gravid mosquitoes to complex chemical blends adds to the already challenging analytical task of not only identifying individual oviposition chemical cues but also the ratio in which they occur.

A recent study by Choo et al. [60] used a reversed chemical ecology approach to identify potential semiochemicals for vector control strategies. The reverse chemical ecology approach is described as an alternative process to identify active semiochemicals by screening for the ligand specificity of olfactory receptor proteins. Olfactory proteins, specifically odour receptor proteins expressed in the antennae of *Cx. quinquefasciatus*, were screened with a panel of 230 odourants for activity. This approach bypassed the often time-consuming traditional bioassay-guided approach to identify semiochemicals from natural sources. Electroantennogram recordings from the antennae of the female mosquito and cage oviposition as well as dual-choice assays demonstrated the potential of acetaldehyde as a potential oviposition attractant over a wide range of concentrations. This was verified by positive results from the newly identified attractant [60]. A better understanding of receptor proteins and the genes that encode them will greatly facilitate the analytical challenge of identifying chemical cues used by mosquito vectors.

Volatile oviposition stimulants could potentially facilitate the selective trapping of female mosquitoes. The challenge lies in the fact that different mosquito species detect and respond to different oviposition stimulant blends. These blends will be unique for each species that needs to be controlled.

### Host-seeking

Takken [18] described the host-seeking behaviour of blood-feeding arthropods as “the orientation to a host from a distance”. Host-seeking generally involves a series of behavioural steps that starts when the insect is activated by a host stimulus and ends when the mosquito lands on a suitable feeding area on the host [18]. Different behavioural cues are used during host-seeking and includes visual cues, long distance chemical stimulants (such as CO_2_, that activates and induces upwind flight), heat and moisture cues that play an important role in behavioural responses in close vicinity of the host, and skin odour cues that influence landing and biting site selection [18, 61–63]. Semiochemical cues used in the host-seeking process are discussed in this section. Emphasis is placed on the importance of carbon dioxide in host finding, the use of host skin volatiles in host selection and/or preference, and lastly the effect of compound blends on host preference.

The use of odour cues during host-seeking behaviour was described by Rudolfs [64] as early on as 1922. Olfaction is now deemed to be the most important stimulus used by mosquitoes during host-seeking [11, 19, 63]. Mosquitoes have very complex olfactory systems containing hundreds of receptor proteins from three different families. As many as 131 and 79 putative odour receptors have been identified in the *Ae. aegypti* and *An. gambiae* genomes, respectively [41]. The receptor families include the olfactory receptors (ORs), ionotropic receptors (IRs) and gustatory receptors (GRs) [41, 65]. The olfactory sensilla that express these proteins are located on the antenna, maxillary palps and proboscis [41].

Sexual dimorphism is present in all the olfactory organs of mosquitoes [9]. The dimorphism correlates with the importance of olfactory cues for female mosquitoes during host location. For example, the females of *An. gambiae* have three to four times more antennal sensilla than males [41]. The neurons housed in these sensilla are thought to express odourant-binding proteins that function specifically for female host location [41]. Further studies on the role of sexual dimorphism in the mosquito olfactory apparatus are needed to clarify how this diversity influences the detection of odour cues during the mosquito life-cycle [9, 41]. Such studies can potentially lead to new discoveries that can enhance our understanding of how odour cues are detected by the different sexes. This could then enable the development of gender specific vector control strategies.

#### The importance of CO_2_

Carbon dioxide is the best-known mosquito kairomone and its role in mosquito host location has been intensively studied [11, 61]. The gas is responsible for priming the flight response and guiding mosquitos to their hosts [11, 19, 61, 66]. Mosquitoes have been shown to respond to very small changes in atmospheric CO_2_ concentrations (~ 0.035%) [12, 67]. Host recognition through changes in CO_2_ levels usually occurs over distances of approximately 10 m when the ambient CO_2_ level changes by the addition of a breath plume containing ~ 4% CO_2_ [12]. A source of CO_2_ is often added to traps as an additional attractant. This can be done by adding dry ice, releasing CO_2_ from gas canisters, or fermenting sugar and molasses in a trap to produce CO_2_. Carbon dioxide substitutes such as 2-butanone can also be added to traps [66, 68–71].

Carbon dioxide is a general cue indicative of the presence of a vertebrate host but mosquitoes do not only rely on CO_2_ because it does not convey information on the potential suitability of the host [11, 19, 61]. There is also variation in how different mosquito species use and respond to CO_2_. Some opportunistic mosquito species, such as *An. arabiensis*, feed on both humans and animals and use CO_2_ as a general host cue whereas other species, such as *An. gambiae*, prefer humans and rely on CO_2_ and other odours that are specific to humans to guide them during flight [19, 66]. The generality of using CO_2_ in host-seeking is thought to increase with the degree of zoophily in mosquito feeding behaviour [61]. Carbon dioxide, by itself, may not be suitable as a chemical lure for selectively trapping vector mosquitoes and in particular not for selectively trapping anthropophilic vector mosquitoes. However, CO_2_ remains an important synergist of host specific chemical cues. Further field work is needed to improve the effectiveness and specificity of CO_2_ as a lure.

Behavioural responses of mosquitoes do not only depend on the presence of CO_2_. Carbon dioxide, human odour and heat was needed to elicit a robust feeding response in wild-type *Ae. aegypti* mosquitoes. The study, by Raji et al. [72] highlighted the importance of considering synergistic chemical compounds, as well as the interaction between chemical and physical cues when studying vector-host interactions. Removing either CO_2_, heat or odour resulted in a reduced feeding response [72]. The intricate combination of both chemical and physical cues that mosquitoes rely on during vector-host interactions complicates the development of artificial lures. This is because chemical lures by themselves do not replicate the physical cues that may be required to elicit the desired behavioural response from the mosquito.

#### Host skin volatiles

Skin volatiles play an important part in host preference for those mosquito species that are specialists on selected hosts [19]. Human skin volatiles have been studied intensively and over 500 compounds have been identified from human skin secretions [73, 74]. Variation in attractiveness between different individuals has been attributed to differences in skin-odour profiles as well as differences in human skin microbial flora [9, 11, 75]. The Knols group provided the first evidence of attraction of highly anthropophilic mosquitoes to a non-human odour source with experiments conducted on Limburger cheese. The smell of Limburger cheese is similar to sweaty human feet. This unique odour was traced back to the coryneform cheese bacteria, *Staphylococcus epidermidis*, that belong to the same genus as the coryneform bacteria found between the toes of human feet. Traps baited with Limburger cheese caught significantly more *An. gambiae* (*s.s.*) than the control traps [11, 76, 77]. A large amount of research has been done since the Limburger cheese experiments confirming the effect of chemical signals derived from skin microorganisms on the host-seeking behaviour in mosquitoes [78].

The intensity and composition of human skin odours is directly related to the type and amount of certain skin bacteria [78]. Skin bacteria metabolise the components of sweat giving sweat its characteristic odour. The volatile chemicals released by skin microorganisms have therefore become a major focal point for studying how mosquitoes distinguish between hosts [78]. These microbially produced volatiles vary between individuals and are thought to aid mosquitos when they need to discriminate between different hosts [78, 79]. A recent study of Busula et al. [79] showed that mosquito species that feed opportunistically also respond behaviourally to a wider array of volatiles. These authors showed that the more opportunistic *An. arabiensis* responds behaviourally to the skin microbiota of all the vertebrate species that it normally feeds on. It was also shown that traps containing bacteria of human origin caught higher proportions of *An. gambiae* than *An. arabiensis* while traps containing bacterial volatiles from chickens caught more *An. arabiensis* than *An. gambiae*. *Anopheles arabiensis* responded equally to all species of bacteria tested whilst *An. gambiae* responded only to four specific bacteria [79]. There is currently a need for further studies on more opportunistic mosquito species as the majority of research regarding skin microorganisms and mosquito behaviour have been done on the more anthropophilic species such as *An. gambiae* [78]. The chemical compounds eliciting the behavioural response from these sources must be identified.

Certain mosquito species prefer human hosts that are already infected with the malaria parasite [19, 80–82]. *Plasmodium falciparum* produces an isoprenoid precursor, (*E*)-4-hydroxy-3-methyl-but-2-enyl pyrophosphate during certain stages of the parasite’s life-cycle, which stimulates red blood cells to increase CO_2_, aldehydes and monoterpene release rates that, when combined, enhance vector attraction and stimulate vector feeding in *An. gambiae* (*s.l*.) [83]. The aldehydes heptanal, nonanal and octanal were detected in larger amounts on individuals infected with the *Plasmodium* parasite. The study, conducted by Robinson et al. [82], revealed an increase in attraction by *An. gambiae* (*s.s.*) towards a synthetic blend containing the above-mentioned aldehydes.

There is evidence that human skin volatiles interfere with CO_2_ detection in mosquitoes. Costantini et al. [84] investigated electrophysiological responses of *An. gambiae* to human-specific sweat components using EAG. The carboxylic acids, (*E*)- and (*Z*)-3-methyl-2-hexenoic acid and 7-octenoic acid were found to reduce the mosquitoes’ response to CO_2_ indicating a possible antagonistic effect. Wind tunnel bioassays conducted by Lacey et al. [62] found that skin volatiles collected from feet onto glass beads override the CO_2_ behavioural responses in *Ae. aegypti* at close proximity to the host. This result confirmed that CO_2_ is involved in long-range activation and is a less important cue for anthropophilic species when they are near the host [62].

Odour cues are also of importance in zoophilic mosquito species. Cooperband et al. [85] reported an activation response in female *Cx. quinquefasciatus* mosquitoes to avian faecal odours. *Culex quinquefasciatus* blood-feeds on birds and is an important vector of the West Nile virus. Headspace volatiles from chicken faeces were collected using SPME whereafter eight volatile aldehydes, namely (*E*)-2-decenal, undecanal, dodecanal, tertradecanal, pentadecanal, hexadecanal, heptadecanal and octadecanal, showed EAG responses when using the antennae (four antennae were used in parallel to elicit an improved response) from *Cx. quinquefasciatus* [85]. The behavioural response of *Cx. quinquefasciatus* to these volatiles still needs to be confirmed.

#### Host skin volatiles and analytical approaches

The vast amount of skin-associated volatiles makes bioassays aimed at determining the behavioural responses of mosquitoes almost impossible. Fortunately, sophisticated analytical techniques are now being applied for elucidating the identity of both specific semiochemicals and potential semiochemical blends. Electroantennogram (EAG) recordings can be used to investigate the antennal responses of mosquitoes to blends of chemicals and single sensillum recording (SSR) studies can be used to identify specific neuron function and type [11]. Gas chromatography coupled to electroantennogram (GC-EAG) recordings can then be used to separate components of blends and investigate their individual electrophysiological activity [11]. Electroantennogram responses are generally measured from the excised head of a mosquito mounted between two glass capillaries containing a saline solution and gold or silver electrode wires. The grounded indifferent electrode is inserted into the back of the decapitated head whilst the recording electrode is connected to the clipped antenna [57, 58, 84, 85].

Comparing profiles of human skin volatiles is not an easy task especially when different sampling methods have been used between studies. The sampling method directly affects how the true chemical profile is represented in the sample. This factor should not be underestimated (see review by Dormont et al. [73]). Sampling methods that have been used include solvent back extraction from a cotton swabs, dynamic headspace adsorption onto various polymers, solid phase microextraction (SPME) [73], and recently (2018), sorptive extraction directly from the skin using polydimethylsiloxane (PDMS) loops that can be worn as bracelets or anklets [86]. Collection of skin volatiles onto glass beads and isolating volatiles from worn nylon stockings and T-shirts have been reported [9, 11, 62, 66, 69, 87]. Dynamic sampling techniques have been developed where the sampled body part is wrapped in a polyvinyl-acetate bag, clean air is then passed over the skin before exiting the bag through a polymer containing filter. The filter is then either thermally desorbed or extracted with a solvent [82]. Some studies attempt to standardise volatile profiles by asking participants to follow particular diets and use specific soaps and shampoos for a time before sampling commences [73]. Such procedures could potentially reduce variation in volatile profiles caused by factors that are unknown.

#### Compound blends

It is not very likely that all skin volatile compounds, with over 500 described in the literature, are simultaneously involved in host-seeking, landing and probing by mosquitoes. Semiochemical cues are rather thought to be specific combinations of a subset of these volatiles. Specific chemical combinations can arise out of combining subsets of only the semiochemicals that occur in the profiles of hosts. For example, it is known that specific compound blends of human skin volatiles are more likely to affect anthropophilic mosquito species such as *Ae. aegypti* and *An. gambiae* [9]. This finding can be rationalised because mosquitoes with different host preferences are more likely to respond to specific blends of volatile compounds that are associated with their preferred hosts [78].

Volatile chemicals emitted by the host can be subdivided into different categories. These include primary odours that do not change when the host diet changes, secondary odours that are dependent on the host diet and environmental factors, and lastly, tertiary odours that come from the application of, for example, lotions and make-up [88]. Verhulst et al. [89] found that the attractiveness of studied volunteers is dependent on the application of skincare products. When volunteers stopped applying a specific product, *An. coluzzi* mosquitoes lost their ability to distinguish between body parts. The skincare product reduced the attractiveness of the skin region where it was applied [89]. This led to the conclusion that skincare products may affect a person’s attractiveness to mosquitoes counter to the expectation that mosquitoes have a preference to bite specific skin regions.

Various studies focussed on developing an attractive synthetic odour blend (i.e. “man-in-a-bottle”) in the past [11]. Van Loon et al. [1] formulated a five-component odour blend for the purpose of mosquito control. The blend was developed by adding two compounds, butan-1-amine and 3-methyl-1-butanol, to the synergistic three component blend of ammonia, (*S*)-lactic acid and tetradecanoic acid which was previously developed by Smallegange et al. [90, 91]. The new blend was found to be even more effective when combined with CO_2_ which increased the attractiveness of the blend synergistically [1]. The slow release of the standard three component synthetic blend with the addition of 3-methyl-1-butanol from low-density polyethylene material attracted more *An. gambiae* than when using the standard blend alone [92]. The challenge in finding the right blend was demonstrated by Smallegange et al. [91]. The synergism between ammonia, lactic acid and carboxylic acids was investigated. Lactic acid alone attracted *Ae. aegypti* but not *An. gambiae*. In contrast, ammonia on its own attracted more *An. gambiae* (the addition of lactic acid did not increase its attractiveness); however, ammonia was only attractive to *Ae. aegypti* in combination with lactic acid. Furthermore, combining ammonia to either lactic or carboxylic acids in a binary blend did not increase the attractiveness of ammonia on its own to *An. gambiae*. The authors also found that the repellent effect of a 12-compound carboxylic acid blend was suppressed with the addition of ammonia [91].

The aforementioned studies demonstrate the vast range of information on mosquito-host volatile chemical cues that have emerged in recent years. Analytical techniques such as GC-EAD and GC–MS have greatly facilitated this process. New analytical methods and novel sampling techniques have made it possible to identify a vast range of compounds potentially useful for vector control strategies. The studies highlight the complexity and analytical challenge in determining the chemical composition of host cues that can change depending on a variety of factors. Chemical cues can originate from bacteria which differ between and within human and animal hosts, or can be influenced by illness or skincare products. Synergistic and antagonistic effects of certain compounds need to be identified. Different mosquito species respond to different chemical compounds and/or blends from hosts differently and this also makes it more difficult to sample and identify effective blends for specific species correctly.

The effectiveness of using odour-baited traps for future vector control strategies was recently demonstrated by Homan et al. [70] in a stepped-wedge cluster-randomised trial. Solar-powered odour-baited mosquito trapping systems (SMoTS), baited with the five-component blend, were installed in households on the Rusinga Island in Lake Victoria, western Kenya. This study showed that malaria prevalence can be reduced by 29.8% in areas where the traps are deployed [70]. The human-biting rate of *Ae. albopictus* in France was reduced to nearly zero with CO_2_ based barrier trap systems [93].

### Sugar-feeding

Kairomones play a key role in locating vital energy sources for flight and other metabolic activities in mosquitoes [11, 94]. Male mosquitoes feed exclusively on carbohydrate sources. In certain mosquito species, the female mosquitoes will take a sugar meal prior to blood-feeding, whilst in other species the female will take a sugar meal after the blood meal [11, 94]. Here we review recent literature on how mosquitoes find these sugar meals with the focus on olfactory cues.

#### Olfactory cues

Sandholm and Price [95] identified the potential of plant volatiles for luring mosquitoes as early on as 1962. These authors observed that certain mosquito species were attracted to light-coloured flowers with distinct odours in the field [95]. Various studies have since been done on the odour mediated sugar source-seeking behaviour in mosquitoes (reviewed by Nyasembe and Torto [96]). These studies revealed that floral odours play an important role for both male and female mosquitoes during sugar feeding [14, 94]. In 1988, Healy and Jepson [97] demonstrated an upwind flight and landing response for *An. arabiensis* and *Ae. aegypti* to the inflorescences of *Achillea millefolium* and *Leucanthemum vulgare*, respectively, during wind tunnel experiments [96–98]. Male and female *An. arabiensis* mosquitoes responded to the inflorescence as well as pentane extracts of *A. millefolium* flowers. A cyclic or bicyclic monoterpene was tentatively identified as the potential active component from these flowers [97].

A study conducted by Gouagna et al. [99] revealed that *An. arabiensis* males are able to discern between potential sugar sources in their environment and that they preferentially feed on plant species that provide the highest metabolic pay off. Field work [100] and laboratory studies, using Y-tube olfactometer assays [100] and two-choice wind tunnel olfactometer bioassays [101], showed that *An. gambiae* (*s.s.*) had a preference for specific plants (namely *Mangifera indica*, *Delonix regia*, *Thevetia neriifolia*, *Senna didymobotrya*, *Senna siamea*, *Cassia sieberiana* and *Parthenium hysterophorus*) [100, 101]. Lahondère et al. [102] demonstrated how orchid odours mediate mutualistic relationships between *Ae. aegypti* and the orchid *Platanthera obtusata*. Monitoring the mosquito’s antennal lobe revealed that both lilac aldehyde and nonanal are detected by mosquitoes. However, the level of these two attractants influence the attractiveness of the orchid to the mosquito. Higher levels of nonanal are released by the orchid species visited by the mosquito, whereas higher levels of lilac aldehyde were released by an orchid not visited by mosquitoes [102]. The identification of the semiochemicals responsible for the behavioural response (i.e. attraction to the sugar source) could potentially lead to new effective synthetic attractants that target both male and female mosquitoes.

Treating natural or artificial sugar sources, i.e. attractive toxic sugar baits (ATSB), with insecticides is another approach employed in vector population reduction programmes [2, 14]. The ATSB technique is limited by the availability of suitable plant products as baits and the non-selectivity towards trapping mosquitoes. This technique relies on the use of ripening fruits, fruit juices and flowers to attract vectors [8]. The limitations associated with the ATSB technique have resulted in the emergence of research aimed at identifying semiochemicals that mediate plant-mosquito interactions and ultimately using these semiochemicals as lures in plant-based vector control strategies [94, 96]. These lures have the potential to be used in surveillance programmes and mass trapping operations, or for contaminating mosquitoes with selective insecticides and entomophathogenic agents such as fungi and viruses [96]. A recent study by Peach et al. [103] found that CO_2_, produced by plants as a metabolite from cellular respiration, works in a multimodal way with olfactory and visual cues to attract mosquitoes to plants. Lures based on plant volatiles need to compete with surrounding vegetation when used in the field and they are often not suitable for long distance attraction of mosquitoes under these conditions. However, by employing plant-based lures the cost concerns of using CO_2_, such as in the form of gas canisters, dry ice, sugar fermentation or propane combustion, and as well as the logistical challenges of transporting CO_2_ into remote areas are eliminated [96].

The use of semiochemicals in vector control strategies was demonstrated by Nyasembe et al. [94] for *An. gambiae*. Six EAD-active plant compounds were identified, *viz* hexanal, β-pinene, limonene, β-ocimene, (*E*)-linalool oxide and (*E*)-β-farnesene [94]. In a follow-up study, the authors used these six compounds as a blend in a plant-based lure. The study revealed that the plant-based lure competed well with the synthetic human odours for trapping malaria vectoring mosquitoes. Furthermore, linalool oxide used on its own or in combination with CO_2_ showed significant potential for use in plant-based odour traps [8]. The challenge in using plant specific kairomones in mosquito lures is substantial. Not only should the preferred sugar source be identified for each mosquito species, but each source should be investigated analytically to identify potential attractants. This presents a huge analytical undertaking due to the chemical complexity present in plant-based samples.

The use of DNA barcoding to profile plant species fed upon by vectors is now possible due to advances in genetic and molecular science. This method utilises specific plant markers to identify plants the mosquitoes previously fed on. These new molecular approaches were used by Nyasembe et al. [104] to identify compounds involved in specific mosquito-plant interactions. Gas chromatography coupled to electroantennogram detector (GC-EAD) recordings were employed to identify odour cues. Unique classes of volatile compounds were detected by vectors from their respective preferred plants. These compounds were benzenoids eliciting a response from *Ae. aegypti*, aldehydes and a benzenoid eliciting a response from *Ae. mcintoshi* and sesquiterpenes and alkenes eliciting a response from *An. gambiae* [104].

The list of attractive volatile chemicals from preferred plants varies considerably between mosquito species and even the compounds that were found to be common across plant species vary in attraction based on differences in the released ratios. The variation is potentially due to an adaptive or innate evolution in mosquitoes that help them to distinguish their plant food source by using specific and general cues in certain ratios [96]. Terpenoids, such as α-pinene, *D*-limonene and β-mycrene are often described as mosquito repellents and are present in several plant families known to be attractive for *An. gambiae* thereby adding to the complexity [105].

The list of known plant attractants and moreover the chemical composition of the plant volatiles remain limited [14, 94, 105]. Plant volatile compounds identified as mosquito semiochemicals can be found in an extensive review by Nyasembe and Torto [96]. These unknowns provide potential study areas and opportunities to implement novel mosquito control and surveillance programmes that are based on plant attractants [94]. Adding to the complexity is the multimodal approach used by mosquitoes to locate sugar sources. Peach et al. [103] concluded that odour, CO_2_ and visual cues are used by mosquitoes to locate inflorescences. Visual cues were deemed more important as an attractant than odour cues alone. Furthermore, the authors showed an overlap of semiochemicals used by mosquitoes for blood-host finding and sugar-feeding [103]. Omondi et al. [106] demonstrated the difficulties of using both plant- and human odour cues together. The authors used a blend of linalool oxide and hexanoic acid that were individually identified as attractive plant- and human cues for *Ae. aegypti*, respectively, but when they were combined a decrease in the amount of *Ae. aegypti* occurred [106]. The frequency of these shared semiochemicals between the two meal types needs further investigation to specifically elucidate differences that mosquitoes use for discrimination. An even greater challenge exists when it becomes necessary to measure variation of semiochemical profiles and to show how these patterns change between preferred plant species and how they are detected by the two sexes of the mosquito. Differences in their responses need to be quantified and the multimodal effect of chemical and physical cues needs to be elucidated. The combination of heat and odour cues appears to function synergistically in the absence of CO_2_, and it is known that a complicated multimodal interaction occurs between CO_2_, human odour and heat when mosquitoes locate their blood host [107]. An improved understanding on multimodal cues can provide a valuable approach to selectively target vector species.

## Conclusions

There is an urgent need to develop new odour-based traps to be used in IVM programmes in order to achieve the ambitious goals set out by the World Health Organisation (WHO) and the complete eradication of malaria goal set by the Bill and Melinda Gates Foundation [5]. To ensure the success of odour-based traps, the focus of lure development strategies using semiochemicals needs to shift to include other types of important vector species, such as *An. arabiensis* and *An. funestus*, as the majority of research to date, has been done on *An. gambiae*. Traps need to be able to target mosquito vectors during different life stages and key behaviours as well as target different sexes while being species-specific. Even though semiochemicals alone might not be adequate as a control tool against mosquitoes, using them in IVM programmes can provide a powerful tool that can help reduce and even eliminate vector populations. Odorants can be used to control mosquitoes by repelling, or by masking human odours, or by attracting mosquitoes with lures into traps. There are, however, substantial shortcomings with current attractants and repellents available to the public. N,N-Diethyl-meta-toluamide (DEET) for example requires high doses, is costly and has an unpleasant smell. Current commercial traps are expensive and bulky as they require a CO_2_ source and usually contain foul smelling synergists. Alternative traps made from recycled materials [108], such as buckets (for example the Gravid *Aedes* Trap) [109] and sticky traps, using products available in the home, such as castor oil [110], need further investigation as these can provide a low-cost and practical solution for vector control in developing countries. The focus of most of the research has primarily been on single olfactory compounds or blends containing less than five compounds. The effect of odour blends, synergistic or antagonistic, on mosquito host selection requires exploring, especially considering that more than 500 different chemicals are associated with human skin of which only a handful will be physiologically active in mosquitoes [65, 73, 74]. Mosquitoes rely on semiochemicals when searching for mates, oviposition sites, sugar meals and blood-hosts. These semiochemicals are potentially valuable for enhancing the selectivity and efficacy of odour-based traps and such traps can form an important part of the IVM strategy. The analytical challenge to find and identify these semiochemicals correctly should not be underestimated and the biologically active blend ratios need to be explored in much greater detail. Successful identification and formulation of these semiochemicals for control implementation will only come from a thorough understanding of the chemical ecology present in each mosquito species’ life-cycle. Such knowledge will facilitate and guide future efforts in the search for these semiochemical cues and signals.

## Data Availability

Not applicable.

## Abbreviations

ATBS: attractive toxic sugar baits
EAD: electroantennographic detection
EAG: electroantennogram
GC-EAD: gas chromatography coupled to electroantennographic detection
GC–MS: gas chromatography–mass spectrometry
GRs: gustatory receptors
IRS: indoor residual spraying
IRs: ionotropic receptors
IVM: integrated vector management
LLIN: long-lasting insecticidal net
ORs: olfactory receptors
*s.l.*: *sensu lato*
*s.s.*: *sensu stricto*
SPME: solid-phase microextraction
SSR: single sensillum recordings
WHO: World Health Organisation
